# Electrical Resistivity Measurements for Nondestructive Evaluation of Chloride-Induced Deterioration of Reinforced Concrete—A Review

**DOI:** 10.3390/ma15082725

**Published:** 2022-04-07

**Authors:** Kevin Paolo V. Robles, Jurng-Jae Yee, Seong-Hoon Kee

**Affiliations:** 1 Department of ICT Integrated Ocean Smart Cities Engineering, Dong-A University, Busan 49304, Korea; kpvrobles@donga.ac.kr (K.P.V.R.); jjyee@dau.ac.kr (J.-J.Y.); 2 National Core Research Center for Disaster-Free and Safe Ocean Cities Construction, Dong-A University, Busan 49304, Korea

**Keywords:** electrical resistivity, corrosion of steel, reinforced concrete, nondestructive evaluation

## Abstract

The objective of this study is to review, evaluate, and compare the existing research and practices on electrical resistivity as a nondestructive technique in evaluating chloride-induced deterioration of reinforced concrete elements in buildings and civil infrastructure systems. First, this paper summarizes the different measurement techniques for gathering electrical resistivity (ER) values on concrete. Second, comparison analyses are performed to review the correlation of ER to different parameters representing corrosive environment and activity of steel corrosion in concrete, such as degree of water saturation, chloride penetration and diffusivity, and corrosion rate. In addition, this research enumerates and individually discusses the different environmental and interference factors that are not related to the electrochemical process of steel corrosion in concrete but directly affect the ER measurements, including temperature, the presence of steel reinforcement, cracks and delamination defects, specimen geometry, and concrete composition. Lastly and most importantly, discussions are made to determine the current gap of knowledge, to improve the utilization of this method in field and laboratory measurements, and future research.

## 1. Introduction

**Figure 1:**
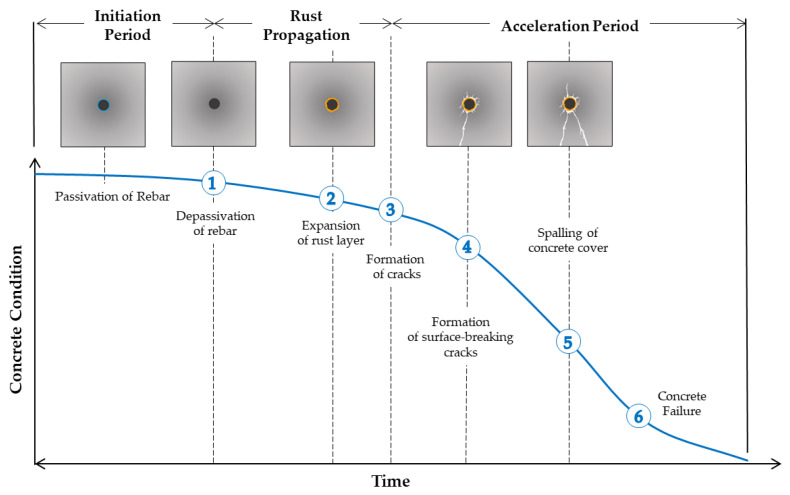
Conceptual illustration of deterioration process of steel corrosion in concrete.

Concrete is considered to be one of the most common and popular construction materials globally because of its high durability, waterproofness, plasticity, and relatively affordable prices [1,2]. However, concrete in buildings and civil infrastructures is susceptible to deterioration from various sources (corrosion of reinforcing steel, freeze–thaw cycles, alkali–silica reaction, acid attacks, exposure to high temperature, etc.). Among them, it has been reported that corrosion of embedded reinforcing steel bars (rebars) is the primary cause of the deterioration of concrete in reinforced concrete structures [3,4,5,6,7,8,9,10,11,12]. Rebar corrosion in reinforced concrete can be defined by multiple phases, starting from the corrosion initiation, rust propagation, and corrosion acceleration (see Figure 1) [13,14,15]. The high alkalinity of concrete (i.e., pH 12~13) produces a thin passive layer (i.e., iron oxide film, Fe_2_O_3_) surrounding the reinforcing steel in sound concrete that significantly reduces the corrosion rate. When either the pH of concrete decreases and reaches a certain level or the chloride ion concentration in concrete exceeds a threshold level, the protective film becomes unstable [16,17]. When the passive layer of the steel reinforcement is unstable and destroyed, it causes the initiation and progression of corrosion in steel. The oxidation of metallic iron causes a 600% increase in the volume of the original iron [18]. The increase in volume will enhance microcracks and internal voids [19], which increase the permeability of concrete, thus accelerating moisture penetration in concrete. When neglected, this can result in serious concrete damages, which can lead to further deterioration of structural elements of reinforced concrete.

Infrastructure corrosion results in significantly high costs in several industrialized countries, globally. In the United States, the annual cost due to corrosion is approximately USD 240 billion. About 20% of these costs are due to the corrosion of reinforced concrete structures such as bridges, highways, tunnels, retaining walls, etc. [20]. Moreover, it is estimated that in Europe, 55% of the repair costs of structures are due to corrosion of steel reinforcement [13]. In addition, the financial loss due to damaged infrastructures caused by steel corrosion is up to 2.9%, 5.2%, and 3.8% of the gross domestic product (GDP) of Korea, the Middle East, and other developed countries, respectively [21,22,23,24]. That is why infrastructure management agencies put importance on determining effective ways to maintain the existing reinforced concrete structures with corrosion issues in order to maintain the structural integrity and increase the service life of structures. This will then significantly decrease rehabilitation costs. Durability provisions and standards have been strengthened and strictly emphasized in different design codes and specifications [25,26]. It is also necessary to conduct condition assessment and early-stage evaluation of structures to monitor the extent of concrete deterioration, check its structural integrity, and establish a detailed diagnosis [27,28].

Accurate monitoring and evaluation of concrete structures is a requirement for proper repair and maintenance [27]. Numerous studies are conducted in evaluating aspects of corrosion related to electrochemistry, such as polarization resistance, corrosion rate, and potential [29,30,31,32,33,34]. Many countries and researchers are focused on the improvement and development of nondestructive test (NDT) methods to assess the durability of concrete. Some of the NDT methods are rapid, safer, and accurate in evaluating corrosion-related parameters. These methods can be classified as follows: visual inspection, electrochemical methods such as electrical resistivity and half-cell potential, polarization resistance, galvanic pulse method, acoustic emission, ultrasonic pulse velocity (UPV), impact echo, and surface wave measurements), ground-penetrating radar (GPR), and optical sensing methods (infrared thermography and fiber Bragg grating) [35]. Zaki et al. [35] summarized and compared the different NDT methods for corrosion monitoring in terms of their advantages, disadvantages, and corrosion evaluation. GPR gives a qualitative analysis of the corrosion damage by analyzing the attenuation of the electromagnetic waves that is affected by the presence of chloride content and corrosion materials [28,36,37,38,39]. Infrared thermography, on the other hand, is conducted through subsurface inspection by detecting the abnormal distribution of the concrete temperature caused by rebar corrosion and internal cracks [40,41,42]. Elastic wave methods are considered more accurate when estimating the effect of corrosion on the mechanical properties of concrete [43,44]. Electrochemical methods—nondestructive evaluation (NDE) techniques—are used to evaluate rebar corrosion in concrete. These kinds of methods need a deeper understanding of all concrete parameters that affect the measurement and assessment of corrosion [45]. In general, these methods give rapid measurements and reliable data for the corrosion rate of steel, the corrosion probability, and the electrical resistivity of concrete [45,46,47,48,49,50]. Examples of electrochemical methods are half-cell potential (HCP), polarization resistance, and electrical resistivity. This paper will focus on the measurement of electrical resistivity and its relation to various parameters related to the electrochemical process of steel corrosion in concrete for evaluating the corrosive environment and activity of steel corrosion in concrete with chloride-induced deterioration. 

Electrical resistivity (ER) of concrete, which has been studied for years [51], is one of the most popular NDT methods for concrete durability evaluation because of the low cost, speed, and simplicity in measurement during laboratory and field applications [52,53,54,55,56]. It is an advantageous method since it measures the ER value of concrete in a nondestructive examination without breaking or damaging the concrete [57]. By definition, ER is a tool for determining the water content and micropore structure of a concrete structure, hence making it effective in evaluating the strength and durability of concrete [55]. It is also an indication of the concrete void (pore volume) and the saturation degree and is used to determine the steel corrosive environment by measuring the concrete resistivity [58,59,60,61]. Moreover, this method is described as the capability of cementitious materials to withstand ion transfer when an electric current is injected into its surface [62,63]. Consequently, ER of concrete has been regarded as a good indicator of the corrosion environment of reinforced concrete elements before the initiation of steel corrosion. In the last twenty years, a research team at Rutgers University in the United States has successfully demonstrated the effectiveness of ER monitoring for the evaluation of the severity of chloride-induced deterioration in existing concrete bridge decks [64,65,66].

In theory, electrical resistivity is the ratio of the electrical potential difference, *V*, over the input electric current, *I*, multiplied by a geometrical constant, *k*. Mathematically, the equation can be written as:

(1) $$\rho =kR=k\left(\frac{V}{I}\right)$$

where *R* is the electrical resistance of concrete. The geometrical constant, *k*, depends on the specimen’s size and shape as well as the device’s electrode (or probe) spacing. Electrical resistivity equipment is usually used to measure laboratory specimens of different shapes, and in the field, it is used on concrete bridge elements such as beams, columns, and slabs.

The main objective of this study is to review, evaluate, and compare the existing research and practices on ER measurements as a nondestructive technique for evaluating chloride-induced deterioration of reinforced concrete elements. This study is composed of four main tasks. First, various measurement techniques to gather electrical resistivity (ER) values of concrete are summarized in the section on measurement techniques. Second, comparison analyses are performed to review the correlation of ER to different parameters related to corrosive environment and activity of steel corrosion in concrete, which include the degree of water saturation, chloride penetration and diffusivity, and corrosion rate. In addition, this research enumerates and individually discusses the different environmental factors and interferences affecting ER measurements that are not directly linked to the consequences of chloride-induced deterioration in reinforced concrete elements, which include temperature, the presence of steel reinforcement, cracks and delamination defects, specimen geometry, and concrete composition. Lastly and most importantly, discussions are made to determine the current gap of knowledge, to improve the utilization of this method in laboratory and field measurements, and for future research.

## 2. Materials and Methods

### Concrete Specimen Preparation

Table 1, Table 2, Table 3 and Table 4 summarize research papers cited in this review. Most of the research used different sizes of concrete cylinders, prisms, cubes, and slabs at either water or lime–water saturation. The specimens were tested at different ages and made with Portland cement, some of which had admixtures such as fly ash and slag cement. The water/binder ratio differed at every specimen. Different methods were used to measure the electrical resistivity of concrete, which will be described in Section 3 in more detail. 

## 3. Measurement Techniques

Several methods have been discovered for measuring electrical resistivity (ER) nondestructively [83]. ER values can be measured using the electrodes placed on the surface of the specimen, positioning an electrode disc or a linear array, or by a four-probe square array on the concrete surface. 

### 3.1. Two-Point Uniaxial Method 

**Figure 2:**
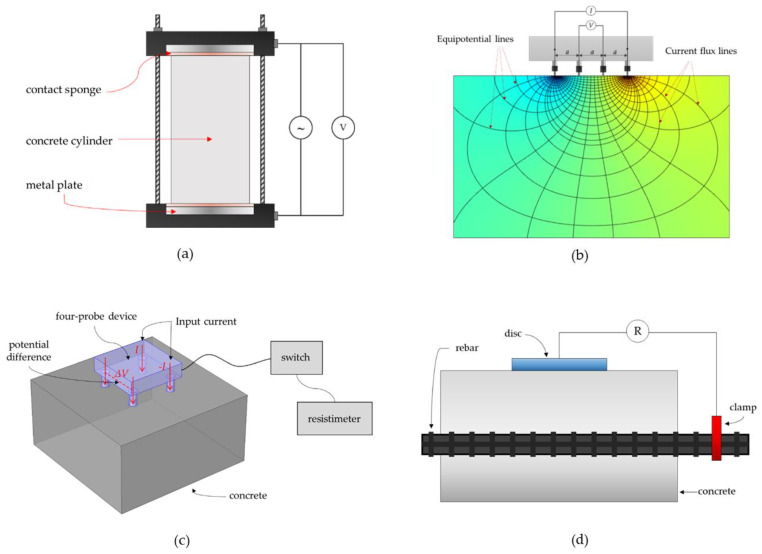
Schematic diagram of the different measurement techniques of electrical resistivity measurements: (**a**) two-point uniaxial method; (**b**) four-point (Wenner Probe) method; (**c**) four-probe square array method; (**d**) electrode–disc method.

In the two-point uniaxial method, also known as the two-electrode method (TEM), two electrodes, usually made by two parallel metal plates, are placed on the surfaces of cylindrical samples (see Figure 2a). Contact sponges are attached at the interface of the concrete and the metal plate to minimize the contact resistance and ensure a good electrical connection. The potential difference between the two electrodes is measured by applying an alternating current (AC). However, it is said that the practical application of this method in the field is very limited because concrete cylinder samples, which are usually taken from existing concrete structures destructively [101], are required in ER measurements [69] using the two-electrode method. 

### 3.2. Four-Point Wenner Probe Method 

The four-point Wenner Probe setup, also known as the Wenner Probe method (WPM), is considered a widely used electrode layout in buildings and civil infrastructure systems, which was initially devised for geological purposes in 1905 [102]. In a Wenner probe configuration, as illustrated in Figure 2b, four probes are placed in a linear and equidistant manner. In this setup, the input current is injected at the two exterior electrodes, and the electrical potential difference is measured through the remaining two interior electrodes [72]. The electrical resistivity of concrete is determined by dividing the applied current by the measured potential as Equation (1). AASTHO TP 95-11 [103] recommended the use of an electrode spacing of 1.5 inch (or 38 mm) with an AC frequency of 13 Hz to measure ER values of concrete. There are several commercially available devices to measure ER values in concrete by the four-point Wenner probe configuration, which conform to the standard specifications of the AASHTO T 358 surface resistivity test method. According to a device’s manual, 10 μA to 200 μA of electrical current are driven to the concrete, depending on the specimen’s contact resistance [104]. The output values are displayed in kΩ-cm, the measurement unit for electrical resistivity.

### 3.3. Four-Probe Square Array Method 

In a four-prove square array method, the measurement device is composed of four electrodes arranged in a square manner with a usual electrode spacing of either five or ten centimeters, as shown in Figure 2c. In this setup, current I is applied to two adjacent electrodes, while the electrode potential difference ∆V is measured through the remaining two electrodes. This method is used by researchers to locate conductive cracks in concrete specimens [105,106,107].

### 3.4. Electrode–Disc Test Method 

Figure 2d shows another method, called an electrode–disc test method, in measuring electrical resistivity. A metal disk is placed on the surface of the concrete to measure the resistance between the electrode, disk, and rebar in the concrete. To convert the calculated resistance into electrical resistivity ρ, the following equation is used:

(2) $$\rho =k\left(R_{disc}-R_{rebar}\right)$$

where $R_{disc}$ is the resistance of the disc, $R_{rebar}$ is the resistance of the rebar, and *k* is the geometrical constant [108]. However, one major disadvantage of this method is that the application is limited to reinforced concrete.

### 3.5. Resistivity Meter through Resistance Measurements

Similar to the electrode–disc method, a couple of researchers determined the electrical resistivity by measuring the electrical resistance and multiplying it by a correction factor [81,91]. The resistance is measured by attaching a resistivity meter to the exposed rebar. However, the specific experimental setup of the devices used by researchers cited in this review is not indicated in their respective papers.

## 4. Correlation of Electrical Resistivity to Various Factors for Steel Corrosion in Concrete

### 4.1. Concrete Degree of Saturation 

The presence of water in concrete pores plays a significant role in the mechanical and durability properties of concrete [109]. An increase in water content could lead to a more vulnerable environment that may result in concrete deterioration. Water in concrete pores serves as an electrolyte in concrete that facilitates the migration of ions in concrete, which is essential for the electrochemical process of steel corrosion in concrete. Therefore, a saturation of concrete is usually considered for evaluation of the corrosive environment of reinforced concrete before corrosion activity initiates. Furthermore, some deteriorations in concrete are manifested as internal cracks due to the expansion of corrosion products (rusts). Such minor concrete defects do not necessarily affect the soundness of the whole structural system. However, these minor defects could increase the permeability of concrete, which allows the concrete to absorb more water or other harmful materials for increasing the corrosive environment (e.g., chloride ion). As such, it can accelerate the deterioration process from corrosion of steel in conjunction with other sources of durability issues such as carbonation, freezing, and thawing. Consequently, the test regions with a relatively higher degree of saturation in concrete are usually interpreted as higher corrosion risk after steel rust propagates in concrete. In addition, a saturation of concrete is one of the most influential factors affecting nondestructive evaluation parameters based on the electrical properties of concrete (e.g., relative permittivity from ground-penetrating radar, corrosion potential from half-cell potentiometer, corrosion current density from electrochemistry-based methods). Therefore, concrete saturation is informed by better interpretation of nondestructive evaluation parameters for comprehensive condition assessment of steel-reinforced concrete subjected to the corrosive environment. 

Electrical resistivity testing is one of the most efficient methods for evaluating the degree of saturation of concrete. A number of studies have informed that the degree of saturation has a major effect on the electrical resistivity measurements [83,110,111,112]. Moreover, published papers argue that for cementitious-based materials, an increase in the degree of water saturation results in a decrease in electrical resistivity [113,114,115,116,117]. To be specific, for concrete specimens, AASHTO TP95-11 indicates that the degree of water saturation has a significant effect in measuring the electrical resistivity of concrete specimens [103]. According to Layssi et al. [69], concrete being a porous material, which can be related to the water content and degree of pore saturation, may exhibit various conductive and insulating characteristics. For example, electrical resistivity is very high in dry conditions, and, for the same concrete, it will be very low in fully saturated conditions [69]. Reichling et al. [118] agree that the electrical resistivity depends on the pore structure and the distribution of the concentration of ions in the liquid. In this perspective, drying the concrete will result in an increasing electrical resistivity. According to Charmchi [110], for saturated specimens put in a 100 °C oven, the largest increase in electrical resistivity happens after 90 min of drying and up to 24 h in the oven. This is, however, not the case for specimens placed at room temperature. 

### 4.2. Chloride Penetration

Chloride penetration can be referred to as how deep the chloride ions present in the environment penetrate the concrete structure, leading to corrosion of such a structure [119]. A number of research papers established that chloride penetration or chloride ingress in concrete may lead to corrosion of steel reinforcement, which is considered one of the major pathologies affecting the durability of reinforced concrete elements [73,120,121,122]. When the chloride ions are charged, the durability of the concrete depends on its capability to withstand the transfer of such ions [62]. The service life of reinforced concrete structures, especially those that are exposed to the marine environment, is also dependent on the permeability of chloride ions [123]. External chloride ions such as deicing salts from highways and bridge decks, seawater in contact with the concrete surface, and chlorides deposited on the surface of the concrete through fine airborne drops that are carried by the wind, are usually the main sources of chloride ions in concrete. The chloride ions enter the concrete by either diffusion of the ions, capillary action, or absorption [124].

In this regard, chloride diffusivity/diffusion coefficient is one of the most commonly used parameters in quantifying chloride ingress/penetration as it controls the transport of chloride ions in saturated concretes [68,72,125]. The diffusion coefficient can be determined through the chloride penetration depth [74]. In order to speed up the measurement of values, researchers either used rapid chloride penetration test (RCPT) [67,69,71,73,77] or rapid chloride migration test (RCMT) [68,73,74,76,123] to determine the diffusivity coefficient. Electrical resistivity (ER) was also used in some papers to determine the diffusion coefficient of concrete specimens [70,72,73,123]. The Nernst–Einstein equation shows the relationship between ER and ion diffusivity [69,70,123,126], as shown in the following equation:

(3) $$D_{i}=\frac{R\cdot T}{Z_{i}{}^{2}\cdot F^{2}}\cdot \frac{t_{i}}{\gamma_{i}\cdot c_{i}\cdot \rho }$$

where *D_i_* is diffusivity for ion *i* [cm^2^/s]; *R* is gas constant (=8.314 J/mol K); *T* is absolute temperature [K]; *Z_i_* is ionic valence *i*; *F* is Faraday constant (=96,500 C/mol); *t_i_* is transfer number of ions *i*; γ_i_ activity coefficient for ion *i*; *c_i_* is ion concentration in the pore water [mol/cm^3^]; and ρ is electrical resistivity [Ωcm].

**Figure 3:**
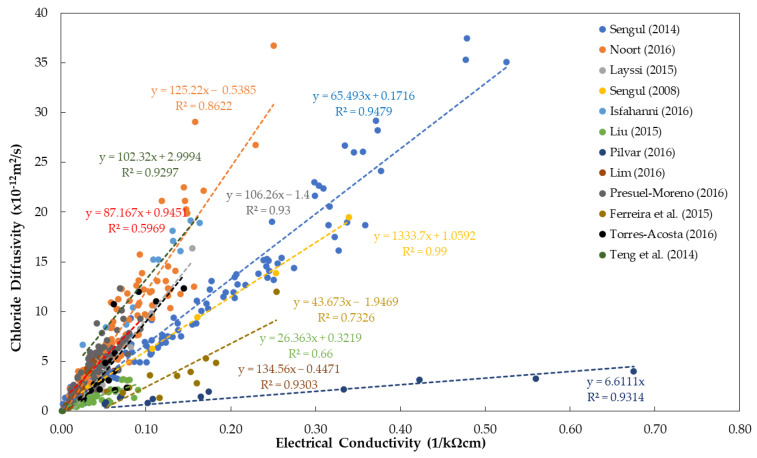
Summary of references showing the relationship between chloride diffusivity and electrical conductivity(1/ER).

In order to visualize the relationship between chloride diffusivity and electrical resistivity, this review paper summarized 11 experimental works in the literature (see Table 1), as shown in Figure 3. In order to present a linear trend, electrical conductivity (which is the inverse of electrical resistivity) is used in the presented graph to show the correlation between the two parameters [67,68,69,70,71,72,73,76]. It can be inferred from the graph that all references show an upward trend in the relationship between electrical conductivity and chloride diffusivity shown on a linear scale. The coefficient of determination (R^2^) of the values gathered from different references ranges from 0.60 to 0.99, denoting a positive correlation. Different methods were used to measure the electrical resistivity of concrete, which will be described in Section 3 in more detail. 

Table 1 summarizes the regression equations R^2^, the material composition, and the experimental condition of the concrete specimens of the cited references.

Table 5 summarizes standards of the relationship between the electrical resistivity and level/magnitude of chloride penetration described in AASHTO [103] and previous research works. Electrical resistivity values are correlated with the risk of chloride penetration with regards to charges passed based on ASTM C1202 [127]. It can be seen that researchers preferred using the standard set by AASHTO, and some opted to make their own standards based on their own experimental setup. It is notable that some researchers used a different range of concrete resistivity values. For example, an ER value of 13 kΩcm denotes a moderate chloride penetration using the AASHTO reference, but the chloride penetration is already high, according to Ghosh et al. [128]. 

### 4.3. Corrosion Rate

The corrosion initiation and activity of steel reinforcement in concrete have been correlated to the severity of deterioration in concrete [134,135,136]. Corrosion rate can be an indicator of the kinetics of steel corrosion in concrete [51,137]. Table 6 summarizes the level of corrosion activity classified by corrosion rate summarized and adapted from different researches [138,139,140]. The corrosion rate of steel is one of the most common parameters used in determining concrete service life models and is used as a durability performance indicator of reinforced concrete structures [141]. The corrosion rate of steel in concrete is simply expressed in terms of charge transfer resistance *R_c_*, the charge transfer resistance involved in the corrosion mechanism, as follows,

(4) $$i_{corr}=\frac{\beta }{\alpha R_{c}}$$

where *α* is the surface area of steel contributed to current flow and *β* is known as the Stern–Geary constant [79,82,142]. Because of the difficulty of directly quantifying the corrosion rate, different electrochemical NDT methods are developed to estimate the corrosion rate by measuring polarization resistance, *R_p_* [138,139]. The *R_c_* value can be determined by subtracting the contribution of bulk concrete, *R_s_*, from *R_p_* as follows,

(5) $$R_{c}=R_{p}-R_{s}$$

**Figure 4:**
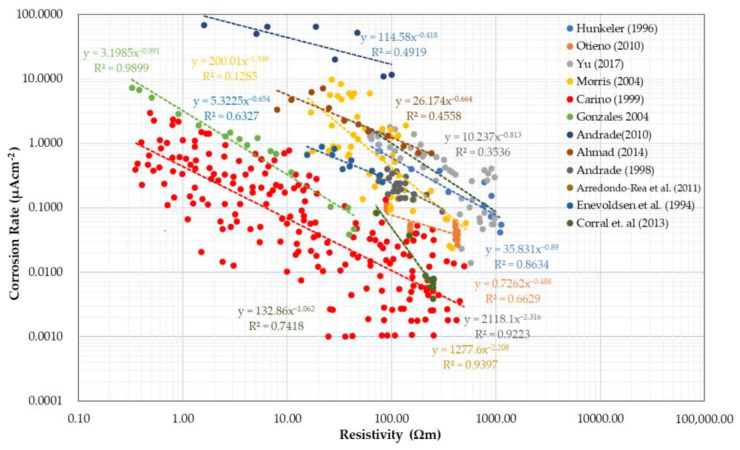
Summary of references showing the relationship between electrical resistivity and corrosion rate.

**Figure 5:**
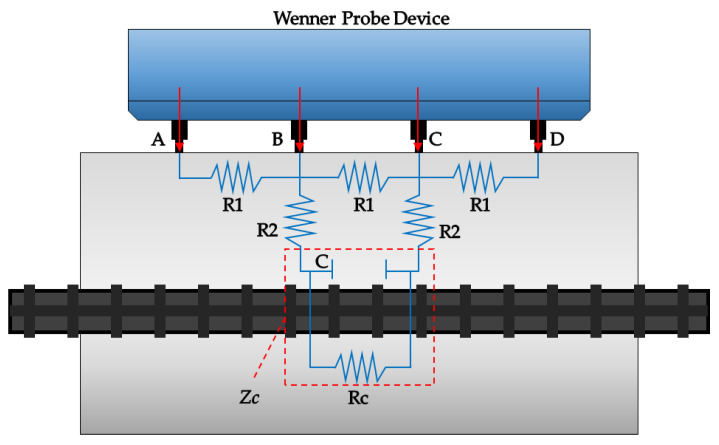
Equivalent electric circuit for electrical resistivity measurement using the four-point Wenner configuration.

Prior researchers demonstrated that the electrical resistivity of concrete is correlated with the rate of corrosion of concrete structures once corrosion of steel in concrete initiates [143,144,145]. ER values can be an indicator of the propagation of steel rusts in reinforced concrete [51,138]. As established by several researchers, the corrosion rate often has an inverse correlation to the electrical resistivity of concrete, as shown in Figure 4. As the value of electrical resistivity increases, the corrosion rate decreases, and a decrease in electrical resistivity results in an increase in corrosion rate [45,78,79,80,81,82,84,85,86,87,88]. All references show a downward trend in the relationship of electrical resistivity and corrosion rate shown in a logarithmic scale due to large variation in values. Statistically, the correlation coefficient of the data gathered from different references ranges from 0.57 to 0.93, denoting a positive correlation. The rationale for the correlation can be explained by the fact that the electrical resistivity values measured over reinforcing steel in concrete are not only affected by concrete properties but also by the geometrical and electrical properties of steel in concrete [146,147,148]. More specifically, several researchers proposed analytical models to explain the effect of corroded steel on the electrical resistivity measurements by the four-point Wenner configuration [149,150,151,152,153]. For example, shown in Figure 5 is an equivalent electric circuit for electrical resistivity measurement using the four-point Wenner probe [152,153]. Theoretically, electrical resistivity measured between the two points B and C in Figure 5, directly over the steel in concrete, is expressed as a combination of electrical resistance of concrete and steel as follows,

(6) ZBC=11R1+1(2R2+Zc)

where *R*_1_ and *R*_2_ are resistance components related to bulk concrete, corresponding to *R_s_*, in Equation (4), and *Z_c_* is frequency-dependent electrical resistance (or impedance) related to electrical properties of steel and steel–concrete interface, which can be expressed as

(7) Zc=11Rc+j2πfC

where *R_c_* is charge transfer resistance, *C* is the capacitance of the electrical double layer in the steel–concrete interface, *j* is the unit imaginary number, and *f* is frequency. Therefore, it can be inferred that the contribution of concrete to electrical resistivity values, which is not really related to the kinetics of steel corrosion in concrete, can explain the large variations in the corrosion rate and electrical resistivity relation from different researchers. As will be discussed in more detail in Section 5, there are various influential factors that affect the electrical resistivity of concrete, such as concrete mixture proportions, clear cover, dry conditions, chloride concentration, etc. Table 2 summarizes important experimental parameters based on concrete specimens with various materials properties and testing methods. 

Nevertheless, under common environmental exposure, corrosion rate has a decreasing relationship with electrical resistivity [59]. Because of practical purposes, a number of research papers, including commercial manuals, presented the general guidelines in terms of corrosion risk when interpreting electrical resistivity measurements [58,105,154,155,156,157,158], as shown in Table 7. Researchers used two different ranges of concrete resistivity values as an indication of the level of corrosion risk. It is observed that a specific value of concrete resistivity may denote a different corrosion risk level, depending on what reference is used. For example, an ER value of 20 kΩcm denotes a moderate corrosion risk for Polder [58] and the instrument manuals, but as per Song and Saraswathy [155], among others, it is still a low-risk level. In addition, Polder and Proceq Manual [104] may have similar ER values as ranges, but in the experimental setup conducted by Polder, concrete cubes were used, whereas Proceq Manual has a default setup of cylindrical samples. The latter, however, introduced a correction factor for different specimen geometries. Moreover, Yu et al. [153] proposed a probabilistic evaluation in assessing the steel corrosion in concrete where the researcher was able to identify the dominant risk in corrosion and determine its probability at different risk levels. This could help in verifying and evaluating the corrosion in rebars, which is usually performed using traditional methods [80]. 

## 5. Other Factors Interfering with the Values of Electrical Resistivity of Concrete

In this section, a summary and comparison of findings in the literature are made on the variation of electrical resistivity values with different factors that are not really related to the electrochemical process of steel corrosion in concrete. It is necessary to consider the effect of such parameters to better interpret the electrical resistivity values for condition assessment of chloride-induced deterioration in reinforced concrete. 

### 5.1. Effect of Temperature of Concrete

**Figure 6:**
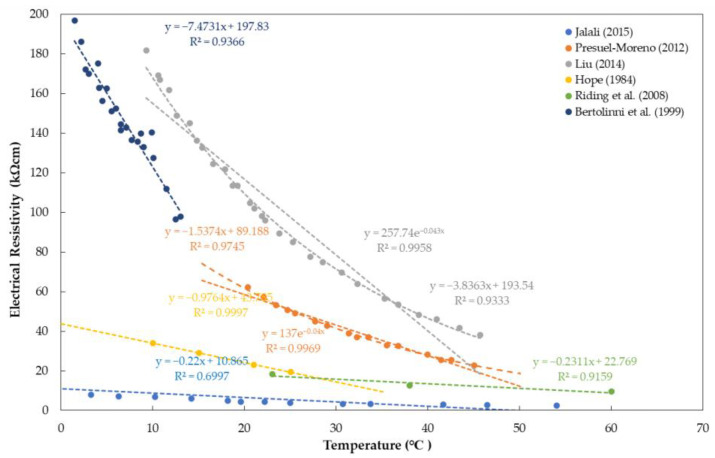
Summary of references showing the relationship between electrical resistivity and concrete temperature.

Several previous research works describe how temperature can influence ER measurements [58,59,62,69,158]. Hornbostel et al. [59] argued that temperature has a minor influence on the measurement of electrical resistivity. However, Azarsa and Gupta [62] mentioned that temperature consideration had played an important role in electrical resistivity measurements on reinforced concrete materials. It is observed from the results from different references (see Figure 6) that ER has an inverse relationship with regard to change in temperature. As the temperature increases, the resistivity decreases, and vice versa [89,90,91,92,93]. As shown in Figure 6 and Table 3, most of the results show a downward linear trend with a linear correlation coefficient ranging from 0.70 to 0.99, concluding that there is a dependency between the temperature and concrete resistivity.

Liu and Presuel–Moreno [75,90], in two of their papers, discussed that the ER value shows an exponential decay as the temperature increases, in contrast to many published studies. As shown in Figure 6, an exponential trend of data published in 2012 shows a correlation coefficient of 0.99, higher than that predicted from a linear trend (=0.97) [92]. The same results are shown in their research published in 2014, where the exponential and linear coefficients of correlation are 0.99 and 0.32, respectively [90]. Moreover, Figure 6 shows that at a certain temperature, the ER values are different at every reference. As written in Table 3, different researchers followed and used different methods in measuring ER of the concrete specimen with various material conditions such as degree of saturation, specimen geometry, etc. It is logically concluded, based on the data gathered, that the difference in mentioned parameters resulted in the variation of ER values.

Previous researchers suggested several ways to compensate for the temperature effect for more consistent ER measurements. First, Presuel–Moreno and Liu [75] suggested that concrete specimens should be fully saturated in order to negate other external environmental parameters in measuring the ER values. In contrast, several researchers [89,90,102] suggested a standard formula that relates the temperature change and electrical resistivity of concrete based on the Arrhenius Law as follows [159,160,161],

(8) $$\rho_{T}=\rho_{0}\cdot exp\left[\frac{E_{a}}{R}\left(\frac{1}{T}-\frac{1}{T_{0}}\right)\right]$$

where $\rho_{T}$ is the measured resistivity at temperature *T* (in Kelvin), $\rho_{0}$ is the measured resistivity at a reference temperature $T_{0}$ (in Kelvin), *R* is the gas constant, and $E_{a}$ is the activation energy for the electrical resistivity. The standard formula can be useful to calibrate the effect of temperature in electrical resistivity measurements. Additionally, a simplified approach to calibrating the effect of temperature was suggested in some research works. For example, Elkey and Sellevold (1995) recommended a three percent (3%) change in the electrical resistivity with every 1 °C change in the temperature. Millard [162] established a correction factor of 0.33 kΩ-cm/°C to compensate for the increase/decrease in temperature. However, standards for correcting ER values based on temperature are yet to be published.

### 5.2. Effect of Presence of Steel Reinforcements

A number of published papers studied the influence of reinforcement steel on the electrical resistivity (ER) of concrete analyzed through experimental and numerical investigation. It is discussed that the presence of rebar in concrete results in disruptions of the electrical current fluxes when an external current is applied [62,147]. Because of the significant effect of the steel on the ER of the specimen, AASHTO TP 95-11 [163] describes that tests on samples with embedded steel should not be valid. From a different perspective, it is concluded in some papers that the presence of rebar in concrete decreases the measured ER. Research pointed out that the alteration of the ER values is brought about by different factors such as the configuration of the electrode, measurement distance, diameter, and spacing of reinforcing steel [143,162,164], among others. Some of the critical factors that affect ER measurements are described in more detail in this section. 

#### 5.2.1. Effect of Configuration of Electrode (Spacing, Direction, and Orientation)

According to Sengul and Gjorv [165], increased electrode spacing results in a larger effect of the embedded steel in the measured ER values. For larger electrode spacings, the researchers obtained an increased value of relative ER (ratio of the ER values measured using the Wenner Probe over the two-electrode method). Similar results were reported by Polder et al. [108]. The change increased in value, however, varies with the direction of the probe. An experimental setup of the slab with steel from Sengul and Gjorv [165] indicates an increase of 33% or a decrease of 25%, depending on the perpendicular or parallel placing of the probes with respect to the orientation of the rebar, respectively [165]. A numerical investigation was conducted by Salehi et al. [166] using nine different configurations of the Wenner Probe with respect to the reinforcing steel. It was concluded that the probe placed perpendicular to the rebar, where the steel is equidistant to the internal electrodes, provides an ER value closest to the measured resistivity value without reinforcing steel in concrete [166].

#### 5.2.2. Effect of Measurement Distance on Embedded Steel 

Polder et al. [108] reported that in extreme cases where four probes are exactly at the top of the reinforcing steel, significantly low resistivity is measured, which is known as a short-cut effect of reinforcing steel in ER measurements. Weydert and Gehlen [164] also reported that ER values decreased to 40% and 60% when it is measured over reinforcing bars with a depth of 10 mm or 20 mm, respectively [109,164]. Furthermore, as concluded by Sengul and Gjorv [165], parallel measurements of probes with respect to reinforcing steel significantly reduced the ER values. These observations explain the reason that the probes should be as far away as possible from the reinforcing steel in concrete.

#### 5.2.3. Diameter of Embedded Steel

Salehi [166], in his numerical investigation, inferred that the rebar diameter does not affect the value of the electrical resistivity. The maximum deviation of five percent was observed in the simulated data. Comparable results and a similar conclusion were stated in the research by Gowers and Millard [143]. In contrast, Lim et al. [146] pointed out that measured ER values decreased with increasing diameter of rebar in concrete but were not able to develop a way of systematically analyzing these differences. Furthermore, other critical effects such as the orientation and location of both electrodes were not fully considered in the research. Therefore, more studies are still needed to obtain general conclusions on the effect of rebar diameters on the electrical resistivity measurements. 

#### 5.2.4. Effect of Concrete Cover

In the numerical investigation conducted by Salehi [166], variations were found in the ER values due to the presence of rebars in concrete when compared with concrete without rebars [167]. Similar to Sengul and Gjorv [165], Salehi [166] correlated the effect to the location of the bars with respect to the electrode spacing, where deeper reinforcement tends to have less influence on the measured values using the Wenner Probe method. Furthermore, Garzon et al. [83], in their simulated model, established a “rebar factor”, which is dependent on contact spacing for laboratory specimens and concrete covers of large concrete. According to the experiment conducted by Sengul and Gjorv [165], the effect of the concrete cover varies with the electrode spacing, considering a configuration of the four electrodes to be directly on top of the rebar.

### 5.3. Effect of Specimen Geometry

The derivation of Equation (1) for the computation of the apparent electrical resistivity considers a semi-infinite and very large medium; therefore, the geometrical composition of concrete can be disregarded [168]. However, in actual laboratory and field applications, the shape and size of concrete are usually defined. A number of papers established that the specimen geometry has an effect on ER values [70,132]. In the research conducted by Sengul and Gjorv [70], it is established that the size and shape of the specimen have a direct effect on electrical resistivity measurements. In the study mentioned, the electrical resistivity of concrete blocks has higher resistivity values than that of the cylinders and cubes due to their larger volume. Both Garzon et al. [83] and Spragg et al. [100] recommended in their papers that corrections should be carried out on specimens with different cross-sectional areas and shapes. Spragg et al. [112], in reference to the paper of Morris et al. [54], recommended a correction factor for considering the effect of the specimen geometry for relatively small concrete specimens. This correction factor considers geometrical parameters such as the sample’s thickness, geometric dimensions, surface area, and probe configuration [169]. For 100 mm × 200 mm concrete cylinders, the correction factor varies from 1.8 to 1.9, using a probe spacing of 38 mm [90]. Morris et al. [137] plotted a correction coefficient graph derived through numerical simulation of varying geometrical sizes. However, this study only focuses on cylinders and has a fixed electrode spacing of 25.4 mm. Other geometric shapes and probe configurations are not investigated. 

For concrete slabs, only limited published papers discussed the effect of its geometric composition on ER measurements. Garzon et al. [83], in their numerical simulation, suggested a shape factor for concrete slabs with undefined sizes. It is also iterated in the research that the rebar configuration affects the value of the shape factor [83]. Gowers and Millard [164], based on their experiment, recommended a reference for ER measurement on concrete slabs: electrode spacing ≤ ¼ slab thickness and lateral dimensions; electrode spacing ≥40 mm and ≤2/3 of the clear cover. Bryant et al. [170] emphasized in their research that for concrete samples with the same concrete composition, the ER of slabs are relatively lower than that of concrete cylinders. Moreover, Chen et al. [55] proposed that the geometrical correction factor should be based on the ratio of the specimen’s length and the device’s probe spacing and the ratio of the specimen diameter and probe spacing for cylindrical specimens. Robles et al. [168], in their experimental and numerical investigation of concrete slabs, concluded that the ratio of the distance of ER probes and electrode spacing affects the ER measurements. Moreover, as per the previous paper by Sengul and Gjorv [70], including the findings of Morris and Moreno [126] and Gowers and Millard [151], overestimations of values are observed for measurements made very close to the edge of the specimen. Gowers and Millard [143] recommended that in measuring the resistivity of the concrete, there should be a minimum distance of twice the contact spacing from the edge of the specimen. 

It is noteworthy that the method of measurement also has an effect on the ER measurements. Using a two-point method, the electric current flows at the whole geometry of the specimen. This is not the case in a Wenner probe configuration, where the electrical resistivity depends both on the specimen geometry and electrode spacing, whereas the larger the spacing of probes, the deeper the current [70]. According to Polder et al. [58], the depth of the current is approximately equal to the electrode spacing, thus making the volume to be tested limited, which affects the measured ER values.

### 5.4. Presence of Defects in Concrete

#### 5.4.1. Surface-Breaking Cracks

The presence of cracks in concrete structures usually indicates that there is a deterioration in the concrete. Chloride ions penetrate the concrete through voids and cracks that can result in reinforcement corrosion [1]. With regards to its effect on the measurement of electrical resistivity, Salehi [167] discussed that there is no systematic study in evaluating resistivity values using the Wenner Probe technique at crack locations. A number of papers discussed that ER could be used to describe, evaluate and detect cracks [171,172,173,174,175]. As per Wiwattanachang and Giao [173], measurements of concrete resistivity are higher at crack zones. However, it was discussed by Salehi [167] that resistivity readings can either be underestimated or overestimated, depending on the type of cracks and the direction of the reading. It was also agreed in a number of papers that the electrode spacing in the Wenner Probe method affects the resistivity measurements of cracks and other concrete irregularities [143,162]. However, Salehi [167] mentioned that changing electrode spacing is only influential to insulated cracks. In addition, it was discussed in some papers that several factors, such as crack depth, type and length of the crack, and probe orientations, can affect the concrete resistivity reading [62,171].

As for crack location, according to Otieno et al. [176], cracks on concrete have no effect on the measured value of resistivity as long as the probes are located away from the locations of the crack. Lataste et al. [171] emphasized that for insulated cracks, the measured resistivity is less than the true resistivity when the measurement is conducted parallel to the crack, whereas it is greater for perpendicular measurements. For conductive cracks, such as insulated cracks, measured electrical resistivity is less than true resistivity in parallel measurements. However, no change was observed when the probe was perpendicular to the crack [171]. Regarding the effect of crack depth and height, Azarsa and Gupta [62] concluded that crack depth has a direct relationship with electrical resistivity, pointing out that as the crack depth increases, the electrical resistivity increases. On the other hand, Shah and Ribakov [174] observed through numerical simulation and experimental studies that measurements of concrete resistivity are higher for insulative cracks and lower for conductive cracks. Similar results are found in the research of Salehi [167] and Lataste et al. [171]. Furthermore, Salehi [167] found that electrical resistivity is insensitive to crack length in the numerical simulation. It was further investigated that the variations in values are just due to the presence of cracks and not the length variations [167].

#### 5.4.2. Delamination Defects 

Currently published papers only explain that the delamination defects cause variation in ER values. However, to the authors’ knowledge, no systematic approach was studied to quantify such ER measurements. Morales [175], in her experimental investigation using various artificial delamination defects and rebar mesh, concluded that the variation in measured concrete resistivities from delaminated zones compared with concrete without delamination is largest in the smallest concrete. Morales [175] also concluded that saturated concrete yields a lower electrical resistivity than dry concrete. [175]. As per Zhao et al. [177], a way to determine the presence of delamination is through the measurement of electrical conductivity. Changes in the electrical resistance of concrete can be related to the detection of delamination. However, no further discussion was made.

Lataste [171], in his experiment, discussed that the average resistivity from concrete health zones is approximately 800 Ωm, while for delaminated zones, the ER values range from 1700 Ωm to 3000 Ωm. Chouteau and Beauliue [172], in their numerical investigation, found that in the presence of delamination in reinforced concrete structures, the measurements of electrical resistivity were different when compared with measurements when delamination is not present. Moreover, in the analytical and experimental investigation conducted by Robles et al. [178], it is discussed that both the size and depth of delamination defects contribute to the variation of ER measurements. When compared with the ER of solid concrete, considering the same square dimension of delamination defects, shallow delamination and deep delamination defects result in higher and lower relative ER values, respectively [178]. In addition, as the size of the delamination defect increases, the relative ER also increases. According to Taillet [105], the best way to measure discontinuities for large concrete structures, concrete resistivity is best measured using DC Current for good quality assessment. It is, however, discussed that using such current instead of AC current may cause unfavorable results due to the polarization effect, which is not considered in the research [105]. 

### 5.5. Concrete Composition

**Figure 7:**
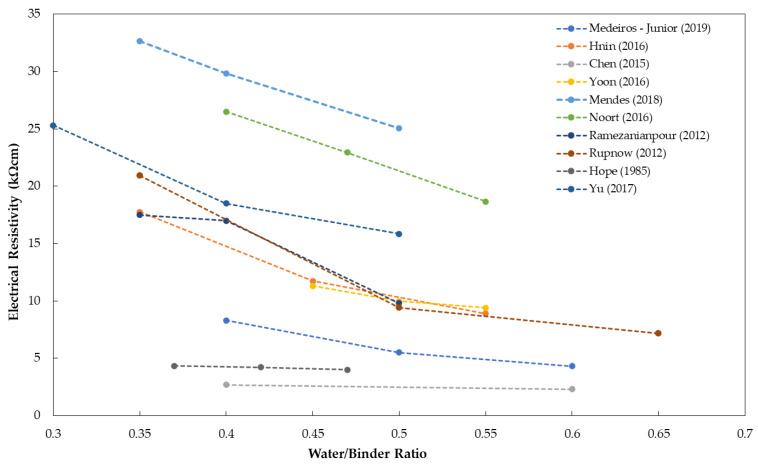
Summary of references showing the relationship between electrical resistivity and water-to-cement ratio.

**Figure 8:**
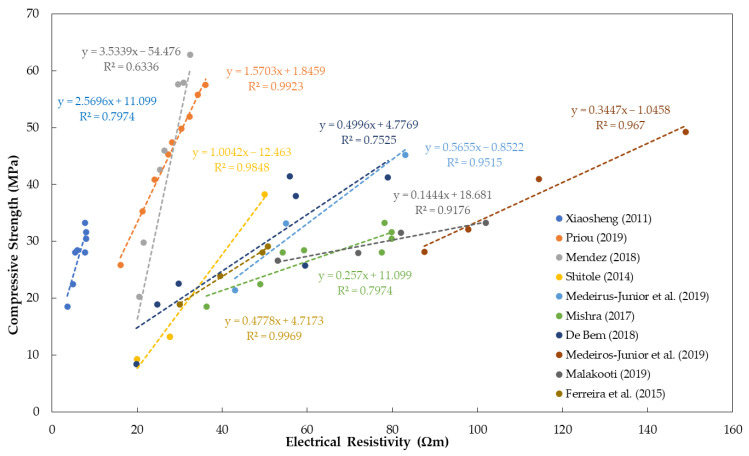
Summary of references showing the relationship between electrical resistivity and compressive strength.

The water-to-cement (or binder) ratio is one of the most important parameters in concrete mixture proportion that influences the mechanical and durability properties of concrete. Numerous researchers concluded that the water-to-cement (w/c) ratio has an inverse relationship to concrete resistivity measurements. Figure 7 shows the relationship between concrete resistivity and w/c ratio, showing the decrease in resistivity measurements as the w/c ratio increases, based on different research articles [51,52,55,68,80,91,97,145,179,180,181]. It is explained that increasing w/c results in an increase in the internal porosity of the concrete, which causes an increase in the permeability of concrete. The increase in permeability is directly connected to the decrease in electrical resistivity [4,68,97,181]. Moreover, increasing porosity (or w/c ratio) leads to decreasing compressive strength of concrete. Consequently, it has been observed by prior researchers that the compressive strength of concrete can be correlated with electrical resistivity [16,59,73]. Studies showed that concrete with higher compressive strength results in lower electrical resistivity and vice versa [96,182]. Figure 8 shows direct proportionality between electrical resistivity and compressive strength [51,76,94,95,96,97,98,99,100,183]. Correlation coefficients of the values gathered from different references ranges from 0.63 to 0.99, denoting a positive correlation [51,76,94,95,96,97,98,99,100,183]. 

While existing standards, measurement procedures, and the majority of published research papers are focused on the electrical resistivity of normal concrete, wide-ranging research has been conducted to analyze the effect of substituting geopolymers to cement without compromising the compressive strength and concrete durability [184,185,186,187]. The motivation of the research is to address the harmful environmental effects of cement, which causes about eight percent of the global carbon dioxide emission [188]. Three of the commonly used geopolymers are fly ash, metakaolin, and slags [189]. In the research of Safari [190], different mixtures of ash and slags exhibit an increasing electrical resistivity with respect to increasing compressive strength over time. It also emphasized that slags exhibit higher electrical resistivity than fly ash when measured at the same age [190]. Moreover, a separate experimental study conducted showed that fly ash-based geopolymers exhibit higher electrical resistivity values than that metakaolin-based geopolymers [189].

In addition, a study showed that bacterial concrete demonstrates higher electrical resistivity values than normal concrete, considering fresh and seawater curing conditions [191]. Similar results are observed in the study of Vijay and Murmu [192]. Considering the studies presented in this review, the relationship of concrete with different admixtures with respect to electrical resistivity still needs analysis and discussion, particularly when compared with the vast number of studies regarding the relationship of electrical resistivity to the compressive strength and other mechanical properties of cement-based concrete.

## 6. Discussion of Findings

Section 4 summarized the current findings on the relationship between electrical resistivity (ER) and various concrete parameters that are related to the electrochemical process of corrosion of steel in concrete. Results gathered from the experiments of multiple researchers for the degree of saturation, chloride penetration, and corrosion rate to ER values are summarized and illustrated in Table 8. Overall, ER value tends to decrease as the degree of saturation or chloride penetration increases. Chloride-included deterioration of corrosion of steel is triggered by a higher concentration of chloride ions around reinforcing steel in concrete than a threshold value. Water in concrete pores facilitates penetration of chloride ions into concrete in the initiation period of corrosion and makes a path of the migration of ions that is necessary for the electrochemical process of steel corrosion in concrete. Therefore, ER measurements are effective for condition assessment of chloride-induced deterioration in reinforced concrete materials. Furthermore, a summary of results in the literature demonstrates that ER values are well correlated with corrosion rate (or corrosion risk): a higher corrosion rate of steel in concrete leads to lower ER values. It can be inferred that ER measurements could be used to evaluate the activity of corrosion after propagation of rust in corroded steel initiates, although there is a lack of a strong theoretical link between ER of concrete and corrosion rate of steel in concrete. 

However, variation in values is still observed in the relationships reported by different researchers in the literature. As described in Section 5, it is well known that ER values are affected by various material and environmental parameters that are not really related to the corrosion process of steel in concrete, as summarized in Table 8. The difference in the values can be attributed to the difference in temperature in ER measurements, various interference effects of reinforcing steel in concrete, specimen geometry and the presence of defects (e.g., surface-breaking cracks and delamination defects), and concrete composition, among others. For example, for the effect of chloride penetration on ER values (see Figure 3), in the study Presuel–Moreno [124], the gathered/tested (R^2^ = 0.60) samples in the paper have different ages, ranging from 90 days to 2 years, whereas, Isfahannni [71], tested all samples (R^2^ = 0.93) after four months at a constant temperature. Table 1 shows that different kinds of binders are used with different water/binder ratios. Regardless, the experiment of Sengul [70], having a linear correlation (R^2^ = 0.99), verified the linear relationship between electrical conductivity and chloride diffusivity by testing a concrete sample at different ages. In addition, for the influence of corrosion rate on ER measurement, Gonzales [82] correlated the resistivity to corrosion rate, having R^2^ = 0.95, by testing a concrete sample at different ages. On the other hand, Morris (2004) [81] (R^2^ = 0.53) used four different kinds of concrete mixtures exposed to the marine environment. Research by Otieno et al. [79] informed that the link between electrical resistivity and the corrosion rate is dependent on the specific concrete and must be established for every individual concrete because of the varying ion components within each concrete [79], which is also attributed to the lack of strong scientific link between ER and corrosion rate as described in Section 4. Correspondingly, the data in Figure 4 show that the relationship between resistivity and corrosion rate showed a scatter plot, which means that further studies and investigations should be carried out to understand its mechanism. 

Therefore, it can be concluded that based on the current studies in the literature, electrical resistivity evaluation is recommendable to be used at a project (or site) level under similar test conditions. Test regions with relatively low ER values can be interpreted as a higher corrosive environment in the initiation period of corrosion and can be further used to estimate corrosion activity and kinetics of steel corrosion in concrete. The latter application could gain more reliability in the context of combined NDE methods such as half-cell potential measurement that more directly informs corrosion activity (or corrosion risk) of steel in concrete. 

Furthermore, it can be concluded that more studies are still needed to consolidate the standard test method for electrical resistivity measurements for the evaluation of chloride-induced deterioration in concrete structures. The standards set by AASHTO [103,193] and commercially available Wenner Probe devices [104] cannot be used directly in all experiments. Several researchers have their own standard in correlating ER to corrosion rate and chloride penetration, as shown in Table 4 and Table 6. AASHTO discussed the proper procedure for measuring the apparent ER in concrete without mentioning the design strength of the concrete. This standard is also limited to concrete cylinders and not to other geometrical shapes. Moreover, the same is observed by the standards set by the Proceq manual in correlating ER to the likelihood and rate of corrosion. Furthermore, the discussion and procedure regarding the effect of the degree of saturation on resistivity values is vaguely mentioned in the manual. It is established in Section 4 that the degree of saturation has a significant influence on the measurement of electrical resistivity; however, a detailed study on this has not been explored. Setting up standards for ER measurements that take into account the effects of corrosion rate, chloride penetration, and degree saturation at different concrete compositions would be a complicated future study but would fill the gap in the existing application of the ER method.

Another interesting finding is that majority of the research and studies cited in this review focused on the influence of one specific parameter (i.e., corrosion rate or chloride penetration) on electrical resistivity values. There are very limited studies that discuss and evaluate the combined effect of two or more parameters on ER measurements. Ahmad [84], in his experimental investigation, discussed that there is a significant difference in the electrical resistivity of corroded reinforced concrete specimens measured with different moisture contents. Robles et al. [178] pointed out in their experiment that instantaneous surface saturation of concrete slabs decreases the effect of delamination defects on concrete resistivity. In addition, it is studied through numerical simulation that the presence of steel reinforcements influences the effect of increasing the dimensions of prismatic concrete specimens [168]. There is a lack of studies on the influence of parameters such as cracks and rebars that are usually present at the same time in reinforced concrete structures. Wenner-probe devices, which are popularly used during field inspections, are very sensitive to many environmental factors. Therefore, special care is needed to accurately interpret the ER values in reinforced concrete structures without clearly knowing the values of critical parameters of ER such as degree of saturation, surface-breaking cracks, delamination defects, and presence and location of reinforcing steel in concrete, as summarized in Section 5. 

It can be derived through the limitations discussed in this review regarding the use of electrical resistivity for the evaluation of chloride-induced deterioration of concrete that ER measurement cannot be a stand-alone method in conducting durability inspections and in acquiring accurate results. Gucunski et al. [64] mentioned that the accuracy of ER in analyzing corrosion-related deteriorating parameters is reported to be “marginally” successful [64] compared with the accuracy of other NDT methods. The limitations of ER measurement motivate a number of studies that supplement and compare ER with one or more NDT methods in durability analysis. Research studies regarding the comparison of Half-Cell Potential and ER have been published [79,121,194,195,196,197]. However, discussions are limited to individual evaluation and do not include a correlation between the two or more NDT methods. Moreover, some researchers discussed and conducted experimental investigations using both ER and ultrasonic pulse velocity, but they are limited to the mechanical properties of sound concrete [198,199,200]. 

In addition to discussion regarding the use of combined NDT methods, recently published papers conducted studies relating to data fusion of multiple NDT data by machine and deep learning to forecast concrete behavior using electrical resistivity and other NDT data [201,202,203,204,205]. The majority of these studies, however, studied the relationship of ER and other NDT methods to concrete properties and mechanical parameters. Torres et al. [206] used Tree Test to predict the tensile strength and ER of concrete. Candelaria et al. [207] predicted the compressive strength of concrete through an artificial neural network, support vector machine, and Gaussian process regression using ER, UPV, and other concrete parameters as input data. Not enough artificial intelligence has been used to correlate NDT data to chloride-induced deterioration. Guzman-Torres et al. [208] performed a multilayer approach in classifying the corrosion risk in concrete using ER, compressive strength, tensile strength, and flexure strength as input data. Still, the study revolved around the corrosion activity and not on the extent of corrosion damage. One interesting study incorporated four NDT methods (ER, UPV, air permeability measurement (APM), and compressive strength using rebound hammer (RH)) to analyze concrete internal damage through random forest-based evaluation [209]. Conclusions made by Chun et al. [209], on the other hand, showed that the predicted results using ER alone are more accurate than using the other three NDT parameters (UPV, RH, and APM). More so, the study focused only on the early stage of corrosion-induced deterioration and did not include an analysis of concrete with further damage, such as surface-breaking cracks and delamination defects.

Another direction that recent studies took related to the use of NDT for concrete durability evaluation is the application of smart technologies such as real-time structural health monitoring and robotics-assisted autonomous measurements. As for real-time structural health monitoring, Kan et al. [210] used an acoustic emission method in real-time monitoring of the fracture behavior of concrete under axial loading. Yu and Caseres [211], Duffo et al. [212], and Taffese et al. [213] used respective self-made sensors embedded in the concrete to monitor real-time corrosion activity by measuring potential corrosion values, carbonation, and chloride penetration. For the application of electrical resistivity, a study discussed the real-time monitoring of ER values to analyze soil properties. For concrete application, another research demonstrated its real-time application in buildings. However, the researchers clarified that ER data gathered have yet to be verified with commercially available devices [214]. The other direction of technological advancement in the field is robotics-assisted autonomous measurement techniques. A research team at Rutgers University developed a robotics-assisted bridge deck inspection tool (RABIT^TM^) in the long-term bridge performance program supported by the federal highway administration in the US (FHWA), which integrates multiple NDT methods (ER, impact echo, GPR, ultrasonic surface waves, and high-resolution computer vision camera). The robotics-assisted technology provides rapid, fully autonomous, and real-time data collection [215,216,217], which enables more reliable, consistent, and rapid data collection on large concrete structures in the field. It can be inferred that currently, the incorporation of smart technology to electrical resistivity (and other conventional NDT methods) for the real-time monitoring and robotics-assisted automated data collection of chloride-induced deterioration of concrete are yet to be fully established. 

## 7. Conclusions

A comprehensive review study was performed to review, evaluate, and compare the existing research and practices on electrical resistivity as a nondestructive technique in evaluating chloride-induced deterioration of reinforced concrete elements in buildings and civil infrastructure systems. Specific findings and conclusions can be drawn as follows:

(1) After conducting an extensive literature review, this study was able to review and evaluate the correlation of electrical resistivity (ER) to critical parameters related to the electrochemical processes of steel in concrete, such as degree of water saturation, chloride diffusivity and chloride penetration, corrosion risk, and corrosion rate. It was observed that ER values showed a downward trend as the value of the three critical parameters increased. Therefore, it can be inferred that ER measurements are effective for condition assessment of chloride-induced deterioration in reinforced concrete materials. Furthermore, ER measurements could be used to evaluate the activity of corrosion after the propagation of rust in corroded steel is initiated, though there is a lack of a strong theoretical link between ER and corrosion damage;
(2) However, variations in values are still observed in the relationships reported by different researchers in the literature. The variations are generally attributed to the fact that ER values are affected by various material and environmental parameters that are not really related to the corrosion process of steel in concrete. This study reviewed and summarized the effects of temperature, presence of rebars, delamination defects, presence of cracks, concrete composition, compressive strength, and specimen geometry, among others;
(3) Therefore, it can be concluded that, based on the current studies in the literature, electrical resistivity evaluation is recommendable to be used at a project (or site) level under similar test conditions. Test regions with relatively low ER values can be interpreted as a higher corrosive environment in the initiation period of corrosion and can be further used to estimate corrosion activity and kinetics of steel corrosion in concrete;
(4) It was also recommended that more studies are needed using electrical resistivity (ER) values to compare the conditions of reinforced concrete structures distributed at national-network levels. This research recommended four directions of future research toward the use of ER evaluation as an NDE tool for network-level application: (1) consolidation of the standard test method for electrical resistivity measurements, (2) more systematic studies to investigate the effects of combined parameters on electrical resistivity measurements values, (3) more study to use the ER in the context of combined NDE methods for comprehensive evaluation of chloride-induced deterioration in concrete structures, in which advanced data fusion techniques by machine learning and deep learning models could be useful, and (4) further developing ER data collection methods using advanced smart technologies such as real-time health monitoring technique and robotics-assisted autonomous data measurements, which enable more reliable, consistent, and rapid data collection in large concrete structures in the field.

In summary, future researchers, engineers, and practitioners can use this review in conducting future studies, experimental investigations, field inspections, and monitoring relevant to the application of electrical resistivity to concrete durability evaluation. The limitations of this method, including its sensitivity to different environmental factors and concrete parameters, should be strictly taken into consideration to make an accurate, logical, and appropriate conclusion.

## Figures and Tables

**Table 1:** Details of specimens used from different references for the correlation of electrical resistivity and chloride diffusivity, including the specimen geometry, regression equation, measurement method, curing condition, age, type of binder, and water-to-cement (w/c) ratio.

Reference	Specimen Geometry	Correlation Equation	R^2^	Method (ER)	Method (Diffusivity)	Curing Condition	Age	Binder	w/c Ratio
[67]	Concrete Disk (100 mm dia. 50 mm thick)	y = 69.12x + 0.49	0.98	TEM	RCPT	Lime–water (20 °C)	3, 7, 14, 28, 90, 365 days	OPC, with other Pozzolanic matls.	0.35, 0.40, 0.45
[68]	Cube (150 mm)	y = 125.22x − 0.54	0.86	-	RCMT	water (20 °C)	7, 14, 27, 28, 56, 91 days	Cement	0.40–0.55
[69]	Cylinder (100 mm dia. × 200 mm)	y = 106.44x − 1.39	0.93	TEM	RCPT	-	-	-	-
				WPM					
[70]	Cube (100 mm)	y = 1333.7x + 1.06	0.99	TEM	RCPT	water (20°C)	3, 7, 14, 28, 90, 180, 365 days	Fly Ash Cement	0.40
	Cylinders								
[71]	Cube (100 mm), (100 mm dia. Core cylinders	y = 102.32x + 3.0	0.93	Computed through conductance	RCPT	water (20–25 °C)	Periodically for 4 months	Cement with Nano Silica	0.50, 0.55, 0.65
[72]	Cylinder (100 mm dia. × 200 mm)	y = 26.363x + 0.32	0.66	WPM	RCPT	Lime–water (21 °C)	28 days	Fly Ash and Slag Cement	0.41
[73]	Cylinder (100 mm dia. × 200 mm)	y = 4.831x + 0.74	0.93	WPM	RCPT, RCMT	Lime–water (23 ± 2 °C),	28, 91 days	PC with Silica Fume	0.35, 0.45
[74]	Cylinder (100 mm dia. × 200 mm)	y = 134.56x − 0.45	0.93	WPM	RCMT	water (25 °C)	7,28, 56, 90 days	OPC, OPC with other Materials	0.25, 0.28, 0.35
	Cube (100 mm)								
[75]	Cylinder (100 mm dia. × 200 mm)	y = 87.167x + 0.95	0.60	TEM, WPM	RCMT	Room and Elevated Temp. (34 °C)	91–100 days, 365 days, 1.5 years, 2 years,	Cement with Slag and silica Fume	0.35, 0.41, 0.47
[76]	Cylinder (100 mm dia. × 200 mm)	y = 43.673x − 1.95	0.73	WPM	RCMT RCPT	Lime–water (20 ± 3 °C),	28 days	Cement with Metakaolin	0.45, 0.60
	Cube(100 mm)								
	Cube (150 mm)								
[77]	Cylinder (100 mm dia. × 200 mm)	y = 104.62x − 1.49	0.68	WPM	-	-	2.5 years	with Microsilica	0.32–0.35
[77]	Cube (100 mm)	y = 104.62x − 1.49	0.68	WPM	-	-	2.5 years	with Microsilica	0.32–0.35

Note: TEM—Two Electrode Method, WPM—Wenner Probe Method, RCMT—Rapid Chloride Migration Test, RCPT—Rapid Chloride Penetration Test, and OPC—Ordinary Portland Cement.

**Table 2:** Details of specimens used from different references for the correlation of electrical resistivity and corrosion rate, including the specimen geometry, regression equation, measurement method, curing condition, age, type of binder, and w/c ratio.

Reference	Specimen Geometry	Regression Equation	(R^2^)	Measurement Method	Measurement Method (Corrosion Rate)	Curing Condition	Age	Type of Binder	w/c Ratio
[78]	-	y = 35.831x^−0.89^	0.65	-	LPR	-	-	-	-
[79]	Beam (100 mm × 100 mm × 500 mm)	y = 0.7262x^−0.488^	0.57	WPM	LPR	NaCl (5%) curing	28, 90 days	PC	0.40, 0.55
[80]	cylinder (150 mm dia. × 300 mm)	y = 132.86x^−1.062^	0.66	WPM	Inverse of ER	-	-	OPC	0.41
[81]	cylinder (15 cm dia. × 22 cm)	y = 200.01x^−1.339^	0.53	Computed using Resistivity meter	LPR		1000 days	Cement	0.40, 0.60
[45]	-	y = 0.4359x^−0.807^	0.64	-	-	-	-	-	-
[82]	Slab (133 × 133 × 7 cm)	y = 3.1985x^−0.991^	0.95	-	LPR	-	-	Cement	0.50
[83]	Slab (160 cm × 140 cm × 10 cm)	y = 114.58x^−0.418^	0.60	Computed using Galvanostat	LPR	-	-	-	-
[84]	Prism (65 mm × 100 mm × 300 mm)	y = 26.174x^−0.664^	0.82	TEM	LPR	NaCl (5%) curing	-	Cement	0.45
[85]	cylinder (75 mm dia. × 150 mm)	y = 10.237x^−0.813^	0.45	-	LPR	water curing	-	OPC	0.50
[86]	cylinder (150 mm dia. × 300 mm)	y = 2118.1x^−2.316^	0.90	-	-	NaCl (3.5%) curing	Monthly (for 6 months)	PC	0.48
[87]	-	y = 5.3225x^−0.654^	0.75	-	-	-	3, 5, 12 months	PC	0.50
[88]	cylinder (150 mm dia. × 300 mm)	y = 1277.6x^−2.208^	0.90	-	-	NaCl (3.5%) curing	Monthly (for 1 year)	Portland Cement	0.48

Note: TEM—Two Electrode Method, WPM—Wenner Probe Method, OPC Ordinary Portland Cement, PC—Portland Cement, LPR—Linear Polarization Resistance using Stern and Geary Formula (1957).

**Table 3:** Details of Specimens used from different references for the effect of temperature on electrical resistivity, including the specimen geometry, regression equation, measurement method, curing condition, age, type of binder, and w/c ratio.

References	Specimen Geometry	Regression Equation	R^2^	Measurement Method	Curing Condition	Binder	w/c Ratio
[89]	50 mm cube	y = −0.22x + 10.865	0.70	WPM	water (20 °C)	-	0.55
[75]	Cylinder	y = −1.5374x + 89.188	0.91	WPM	water	PC with FA	0.41
[90]	Cylinder	y = −3.8363x + 193.54	0.93	WPM	water	PC with FA	0.41
[91]	25 × 25 × 100 mm prism	y = −0.9764x + 43.785	0.99	Computed through Resistance Values	-	Normal PC	0.37, 0.42, 0.47, 0.57
[92]	Cylinder (100 mm dia. × 200 mm)	y = −0.2311x + 22.769	0.92	-	-	PC with FA	0.32–0.53
[93]	100 × 100 × 300 prism	y = −7.4731x + 197.83	0.94	-	-	OPC	0.45, 0.65

Note: WPM—Wenner Probe Method, OPC—Ordinary Portland Cement, PC—Portland Cement, FA—Fly Ash.

**Table 4:** Details of Specimens used from different references for the correlation of electrical resistivity and compressive strength, including the specimen geometry, regression equation, measurement method, curing condition, age, type of binder, and w/c ratio.

Reference	Specimen Geometry	Regression Equation	R^2^	Measurement Method	Curing Condition	Age	Binder	w/c Ratio
[94]		y = 2.5696x + 11.1	0.79	-	water (23 ± 2 °C)	7, 28 days	OPC	0.55
[95]	Slab/beam/cylindrical sample	y = 1.5703x + 1.85	0.99	WPM	water	-	PC	0.40, 0.45, 0.50
[51]	cylinder (100 mm dia. × 200 mm)	y = 3.5339x − 54.48	0.63	WPM	-	28 days	PC with admixtures	0.32–0.72
[96]	150 mm cube	y = 1.0042x − 12.46	0.98	WPM	-	3, 7, 28 days	OPC, some samples with silica fume	0.5
[97]	cylinder (100 mm dia. × 200 mm)	y = 0.5655x − 0.85	0.95	WPM	water (23 ± 2 °C)	28 days	PC	0.42, 0.48, 0.54, 0.60
[98]	100 mm cube	y = 0.257x + 11.10	0.8	-	-	3, 7, 28 days	PC with slag	0.55
[93]	cylinder (100 mm dia. × 200 mm)	y = 0.4996x + 4.78	0.75	WPM	-	28 days	PC with limestone	-
[99]	cylinder (100 mm dia. × 200 mm)	y = 0.3447x − 1.05	0.97	WPM	water (23 ± 2 °C)	3, 7, 28 days	PC	0.42, 0.48, 0.54, 0.60
[100]	cylinder, prism	y = 0.1444x + 18.68	0.92	WPM	-	7, 14, 28, 56 days	Different Cement type	0.44
[76]	Cubes and Cylinders	y = 0.4778x + 4.72	0.99	WPM	water (20 ± 3 °C)	7, 14, 28, 90, 180 days	Cement with admixtures	0.45, 0.60

Note: WPM—Wenner Probe Method, OPC—Ordinary Portland Cement, and PC—Portland Cement.

**Table 5:** Range of electrical resistivities at a different level of chloride penetration.

	Chloride Penetration Levels According to Electrical Resistivity of Concrete [KΩ CM]					
References:	Very High	High	Moderate	Low	Very Low	Negligible
[52,104,129,130]	–	<12	12–31	21–37	37–254	>254
[131]	–	<7	7–13	13–24.3	24.3–191	>191
[62]	–	<6.7	6.7–11.7	11.7–20.6	20.6–141.1	>141.1
[132]	–	<16	16–28	28–50	50–343	>343
[101]	–	<5	5–10	10–20	20–200	>200
[133]	<5	5–7.5	7.5–15	15–35	>35	–

**Table 6:** Corrosion risk levels classified by the corrosion rate of steel in concrete [138,139,140].

Corrosion Risk Level	Corrosion Rate [μAcm^−2^]
Passive/very low	<0.2
Low/moderate	0.2–0.5
Moderate/high	0.5–1.0
Very high	>1.0

**Table 7:** Range of electrical resistivities at different corrosion risk levels.

	Corrosion Risk According to Electrical Resistivity of Concrete (kΩ⋅cm)			
References	High	Moderate	Low	Negligible
[58,154,158]	<10	10–50	50–100	>100
[155,156]	<5	5–10	10–20	>20
[104,157]	≤10	10–50	50–100	≥100

**Table 8:** Summary of findings and research gap with regards to the relationship of electric resistivity to various environmental factors and parameters.

Parameter	Relationship to ER	Research Gap
Degree of Saturation	Inversely proportional to ER values	Not enough experimental analysis in quantifying the interference of degree of saturation to ER measurements in concrete. Given discussions are general and vague.
Chloride Ion Penetration	Inversely proportional to ER values in a linear scale	Variations in ER values from different studies are observed. Other parameters are not considered/controlled in the analysis of data
Corrosion Rate	Inversely proportional to ER values in a logarithmic scale	Variations in ER values from different studies are observed. Other parameters are not considered/controlled in the analysis of data There is a lack of studies regarding the relationship between ER and corrosion level and the extent of damage to concrete. Evaluation of corrosion in steel using electrical resistivity with other kinds of NDT such as half-cell potential is recommended. Discussions focused on saturated conditions; evaluation at different degrees of saturation should be further studied.
Temperature	Inversely proportional to ER values in both linear and exponential scale	Considering the discussions made, no unified conclusion and relationship are shown with respect to ER and temperature of concrete.
Presence of Steel Reinforcement, Electrode Spacing and Configuration, Presence of Cracks, and Presence of Delamination Defects	Variations in ER values are observed with respect to the mentioned parameters	Not enough studies discussing a systematic approach to measuring ER with respect to the presence of the mentioned parameters. Given discussions are vague and do not include analyses of how to quantify the influence of the parameters on ER measurements.

## Data Availability

Data are contained within the article. However, the data presented in this study are also available on request from the corresponding author.
